# Infectious Complications of Targeted Therapies in Children with Leukemias and Lymphomas

**DOI:** 10.3390/cancers14205022

**Published:** 2022-10-14

**Authors:** Ioannis Kyriakidis, Elpis Mantadakis, Eftichia Stiakaki, Andreas H. Groll, Athanasios Tragiannidis

**Affiliations:** 1 Department of Pediatric Hematology-Oncology & Autologous Hematopoietic Stem Cell Transplantation Unit, University Hospital of Heraklion & Laboratory of Blood Diseases and Childhood Cancer Biology, School of Medicine, University of Crete, 70013 Heraklion, Greece; 2 Department of Paediatrics, Paediatric Hematology/Oncology Unit, Faculty of Medicine, Democritus University of Thrace, 68100 Alexandroupolis, Greece; 3 Infectious Disease Research Program, Center for Bone Marrow Transplantation and Department of Pediatric Hematology and Oncology, University Children’s Hospital Münster, D-48149 Münster, Germany; 4 Pediatric and Adolescent Hematology-Oncology Unit, 2nd Department of Pediatrics, School of Medicine, Faculty of Health Sciences, Aristotle University of Thessaloniki, AHEPA Hospital, 54636 Thessaloniki, Greece

**Keywords:** bacterial infections, virus diseases, invasive fungal diseases, antibodies, monoclonal, immune checkpoint inhibitors, CAR T-cell therapy, hematological malignancies, leukemia, lymphoma, child

## Abstract

**Simple Summary:**

Targeted therapies in children with hematological malignancies moderate the effects of cytotoxic therapy, thus improving survival rates. They have emerged over the last decade and are used in combination with or after the failure of conventional chemotherapy and as bridging therapy prior to hematopoietic stem cell transplantation (HSCT). Nowadays, there is a growing interest in their efficacy and safety in pediatric patients with refractory or relapsed disease. The compromised immune system, even prior to therapy, requires prompt monitoring and treatment. In children with hematological malignancies, targeted therapies are associated with a comparable incidence of infectious complications to adults. The exact impact of these agents that have different mechanisms of action and are used after conventional chemotherapy or HSCT is difficult to ascertain. Clinicians should be cautious of severe infections after the use of targeted therapies, especially when used in combination with chemotherapy.

**Abstract:**

The aim of this review is to highlight mechanisms of immunosuppression for each agent, along with pooled analyses of infectious complications from the available medical literature. Rituximab confers no increase in grade ≥3 infectious risks, except in the case of patients with advanced-stage non-Hodgkin lymphoma. Gemtuzumab ozogamicin links with high rates of grade ≥3 infections which, however, are comparable with historical cohorts. Pembrolizumab exhibits a favorable safety profile in terms of severe infections. Despite high rates of hypogammaglobulinemia (HGG) with blinatumomab, low-grade ≥3 infection rates were observed, especially in the post-reinduction therapy of relapsed B-acute lymphoblastic leukemia. Imatinib and nilotinib are generally devoid of severe infectious complications, but dasatinib may slightly increase the risk of opportunistic infections. Data on crizotinib and pan-Trk inhibitors entrectinib and larotrectinib are limited. CAR T-cell therapy with tisagenlecleucel is associated with grade ≥3 infections in children and is linked with HGG and the emergence of immune-related adverse events. Off-label therapies inotuzumab ozogamicin, brentuximab vedotin, and venetoclax demonstrate low rates of treatment-related grade ≥3 infections, while the addition of bortezomib to standard chemotherapy in T-cell malignancies seems to decrease the infection risk during induction. Prophylaxis, immune reconstitution, and vaccinations for each targeted agent are discussed, along with comparisons to adult studies.

## 1. Introduction

Despite improvement in the overall cure rates of childhood leukemias and lymphomas, outcomes following conventional chemotherapy and HSCT have reached a plateau. Refractory and relapsing (r/r) disease is still a major problem, along with treatment-related toxicity. Beyond genetic alterations, precision medicine features, such as immunophenotype, drug response, and residual disease, allow better stratification and prognostication of the disease and offer new insights into therapeutics through the implementation of targeted therapies [1,2]. Targeted therapies differ from conventional cytotoxic therapies in their specificity towards targeted pathways, that can compromise the growth and survival advantage of cancer cells rather than damaging indiscriminately all rapidly dividing or irradiated cells. Targeted therapies comprise biologics (mainly monoclonal antibodies or mAbs) and small-molecule drugs, and they have evolved significantly since their first members were initially approved for the treatment of blood cancer (rituximab in 1997 and imatinib in 2001, respectively). Not until the last decade, pediatricians and hematologists have gained considerable experience with their use in the pediatric setting, although their use is still not widespread. While most of these agents seem to be generally well tolerated and safe, prompt monitoring for the recognition and treatment of infectious complications should be conducted. Nevertheless, the assessment of the exact contribution of targeted therapies to infection rates in children with hematologic malignancies is problematic, as their immune system is compromised because of their disease and the prior use of conventional chemotherapy. Additionally, preceding and concomitant immunosuppressive treatments also affect the infection rates observed [3,4]. The aim of this review is to summarize and update our current knowledge of the infectious complications following molecularly targeted therapies and immunotherapy in the context of treating childhood leukemias and lymphomas. The mechanisms by which these agents affect the immune system are scrutinized and pooled analyses of results are presented where applicable. Table 1 describes all relevant Food and Drug Administration (FDA) and European Medicines Agency (EMA) approved targeted therapies for pediatric patients.

## 2. Monoclonal Antibodies

### 2.1. Anti-CD20 mAb

Rituximab is a first-generation anti-CD20 mAb that displays a terminal half-life of 18 to 32 days, depending upon the dosing scheme. After its approval, two more generations of anti-CD20-targeted agents have emerged: second-generation humanized or fully human mAbs to reduce immunogenicity and improve efficacy, and third-generation mAbs bearing an engineered Fc region to augment complement-dependent cytotoxicity (CDC) and antibody-dependent cell-mediated cytotoxicity (ADCC) [16]. CD20 is crucial for B-cell development and differentiation and is expressed on both normal and malignant B-cells, beginning at the pre-B phase, and progressively increasing in concentration until B-lymphocyte’s maturation. Interestingly, neither early pro-B-cells nor plasma cells express CD20, so rituximab cannot impair immunoglobulin production directly [4]. However, subsequent courses of treatment with rituximab seem to lead in HGG, especially in children, which seems to last for 5 to 12 months and may require immunoglobulin substitution in selected cases [3,17]. A large retrospective study on 211 pediatric and adult patients with non-Hodgkin lymphomas (NHL) who received rituximab found that HGG occurs in 38.5% of patients with normal baseline IgG levels. The risk was greater in patients who received maintenance rituximab, and symptomatic HGG that necessitated intravenous immunoglobulin (IVIG) administration developed in 6.6% of patients, suggesting that the monitoring of serum IgG in all patients receiving rituximab is necessary [18]. CD20-positive B-cell lysis is prompted by various mechanisms: CDC, ADCC, antibody-dependent cellular phagocytosis (ADCP), induction of apoptosis via programmed cell death, lysosome-mediated non-apoptotic death triggered by homotypic adhesion, and reactive oxygen species-mediated death through NADPH (nicotinamide adenine dinucleotide phosphate) and, eventually, via sensitization to chemotherapy [4,19]. Recent studies suggest the implication of CD20 in B-cell receptor (BCR), CXCR4/CXCL12, and IL4/STAT6 signaling pathways, which in turn implies that the immune effects of rituximab are not restricted to the depletion of CD20-positive B-cells [20,21]. Rituximab treatment leads to rapid (within 3–7 days) and profound (≥90%) depletion of CD20+ cells along with depletion of CD3+ CD20+ cells (3–5% of T-cells) that lasts for about 12 months. In addition, rituximab affects the function of B-cells as antigen-presenting cells (APCs), increasing the immature and transitional B-cells, thus leading to a dysfunction of CD4+ and CD8+ T-cell responses. Anti-CD20 blockade also affects Th17+ cells, which are responsible for the protection of mucosal barriers and contribute to pathogen clearance at mucosal surfaces. Moreover, late-onset neutropenia that occurs 1 to 5 months after treatment has been reported in 5–15% of patients receiving rituximab. It usually lasts for months but is not associated with a significant increase in infection rates and usually resolves spontaneously [3].

In the context of infectious complications, rituximab has a boxed warning for hepatitis B virus (HBV) reactivation and progressive multifocal leukoencephalopathy (PML) by the John Cunningham (JC) virus (human polyomavirus 2) [5,16]. In general, severe infections are expected in 4% of patients undergoing rituximab monotherapy and this percentage rises with concomitant chemotherapy (30–50%) [5,22]. Large pharmacoepidemiologic and retrospective cohort studies have confirmed the relatively low grade ≥3 infection rates (18.2% to 42.5%) and low infection-related mortality rates within a year post-rituximab, compared with the risk conferred by conventional chemotherapy and the underlying disease alone [23,24,25,26,27]. Table 2 illustrates these differences based on published randomized controlled clinical trials (RCTs). The overall severe infection incidence rate was calculated at 13 per 100 patient-years, while the use of rituximab in pediatric oncology patients confers a 3.5-fold increase in the risk of infection (95% CI: 1.37 to 9.00; p_adj_ = 0.009). Regarding immune reconstitution, B-cell count recovery has been achieved at a median of 9 months after rituximab. The time to recovery of CD19+ CD27+ memory B-cells lasts longer (a median of 15.7 months), posing a threat to the success of routine childhood vaccinations. The serum IgM and IgG levels drop in a significant proportion of patients following rituximab infusion and this can last beyond 12 months (40.8% and 33.9% vs. 23.2% and 13.7%, respectively) [23]. As compared with other children treated with rituximab, fatal infections were more prevalent (14.3%) among allo-HSCT recipients, whereas in utero exposure to rituximab is associated with septic episodes in newborns [26,28]. Notably, the observed infections were attributed predominantly to sepsis along with less frequent but severe infections due to cytomegalovirus (CMV), adenovirus, herpes simplex virus (HSV), varicella-zoster virus infection (VZV), influenza virus, Epstein-Barr virus (EBV), and invasive fungal diseases (IFDs), such as *Pneumocystis jirovecii* pneumonia (PJP) [23,24,25,26,27].

The phase 3 NCT01516580 trial that recruited 164 children with high-risk mature B-cell NHL who received standard LMB chemotherapy with or without rituximab, reported no significant increase in grade ≥4 infections in patients who received rituximab compared with the chemotherapy-only arm (18.5% vs. 11.1%; grade 3 infection in 40.1%, and all-grade infection in 58.6% of children under rituximab). This trial confirmed the significantly higher proportion of patients with HGG in the LMB-rituximab arm at the end of therapy (70.3% vs. 46.8%; *p* = 0.0019) and 1 year after treatment (55.9% vs. 25.4%; *p* = 0.0002). The most common infections in the rituximab arm were sepsis (17.3%), central venous catheter-associated infections (13%), lower respiratory tract infections (11.7%), and enterocolitis (8.6%) [29]. Pediatric B-NHL regimens, such as the LMB, exhibit higher acute infectious toxicity than DA-EPOCH-R, an adult regimen. The Inter-B-NHL ritux 2010 study reported grade ≥3 infections in 4% of the courses and 17% of the patients compared with 58.6% infections grade ≥3 in other LMB-based chemotherapy trials. The LMB2001 trial included 42 children with primary mediastinal B-cell lymphoma (PMBCL) treated with LMB with or without rituximab, and recorded one case of candidemia (4.8%) in the rituximab arm, but no toxic death [30]. Strikingly, a study on 88 children with diffuse large B cell lymphoma (DLBCL) that included patients with human immunodeficiency virus (HIV) infection did not observe an increased incidence of infection-related deaths in patients who received rituximab plus chemotherapy compared with those who were treated with chemotherapy alone [31].

In terms of infectious complications, the B-NHL 2004 M and B-NHL-2010 M trials utilized reduced-intensity chemotherapy plus rituximab in children with Burkitt lymphoma (BL)/B-cell acute leukemia (B-AL), DLBCL, and mature B-cell lymphoma, and both reported no rituximab-related severe infections, while the serum IgG levels recovered in half of the patients within a year of treatment [32,33]. The NCT00324779 phase II window trial on rituximab monotherapy in 136 newly diagnosed children with CD20+ NHL and B-AL reported grade ≥3 infections in four cases (2.9%) and an infectious severe adverse event within 30 days after rituximab administration (days 19–25), but after first course of B-NHL-BFM 04 protocol [34]. Another pediatric study on r/r BL/B-AL after BFM-based first-line therapy attributed one rituximab-related death (1.7%) to *Candida glabrata* sepsis [35]. The addition of rituximab to the revised NHL-BFM-95 protocol for 57 children with BL did not result in significantly different rates (61.2%) of grade ≥3 infection [36].

High grade ≥3 infection rates have been documented in the CCCG-BNHL-2015 report that involved 419 pediatric patients with de novo aggressive mature B-NHL: 54.1% to 64% and one fatal case of measles in the rituximab arm [37]. Another trial that assessed the safety of rituximab addition to the FAB/LMB 96 chemotherapy regimen in 45 children with stage III/IV B-NHL reported no toxic deaths, while it recorded one rituximab-related case of grade 3 infectious colitis (2.2%), and estimated the rate of grade ≥3 infections at 41%, 26%, 2%, and 14% during the first induction, second induction, first consolidation, and second consolidation phases, respectively [38]. Similarly, a study on 20 children with r/r CD20+ NHL and mature B-ALL undergoing R-ICE reported grade ≥3 infections in 30% of the cohort, but no fatal infections [39]. Despite the anticipated higher infection rates in children treated with rituximab in combination with chemotherapy for the central nervous system (CNS)-positive BL/B-AL, recent trials (including ANHL01P1) suggested otherwise. Grade ≥ 3 infections occurred in 39–63% and fatal infections occurred in 2.5–6.4% of the patients during all treatment phases [40,41]. Regarding de novo primary CNS lymphoma in pediatric patients treated with rituximab plus methotrexate and temozolomide, the CALGB/Alliance 50202 study reported that only 7% of patients developed a grade ≥3 infection vs. 18% of patients in the high-dose arm that included consolidation with etoposide plus cytarabine [42].

No significant increase in the infection risk has been observed between de novo CD20+ pediatric patients with ALL treated with chemotherapy supplemented by rituximab, while the addition of bortezomib in the same population also seems to minimally affect the toxicity profile (2.9% with fatal infection; 67.6% with grade ≥3 infection; invasive fungal infections (IFDs) in 40% of episodes) [43,44]. In contrast, the addition of rituximab to a modified hyper-CVAD regimen for CD20+ pediatric patients with ALL was associated with a higher rate (9.2%) of fatal infections [45]. With regard to children with follicular lymphoma, the administration of R-CHOP was associated with a favorable safety profile in terms of infections [46]. Of interest, in a phase II study of r/r DLBCL that included some children, the combination of rituximab plus brentuximab vedotin (BV) showed no increased infection risk compared with BV monotherapy (12.6% vs. 9%) [47].

In summary, the available data suggest a subtle increase in infectious risks in association with rituximab use in children with lymphoma and/or leukemia. The oncologic diagnosis alone confers a high risk of developing grade ≥ 3 infections after a single course of rituximab (HR 6.25; 95% CI: 1.52–25.68; p_adj_ = 0.01), even though the rates of severe infections are lower for rituximab compared to other immunosuppressants [23]. Regarding PJP prophylaxis with trimethoprim/sulfamethoxazole for those receiving rituximab, consensus documents suggest that it should only be considered for patients under concomitant therapy with 0.4 mg/kg/day or 16 mg/day of prednisone or its equivalent for at least four weeks, for patients receiving anti-CD20 mAb every 14 days, and for those with underlying diseases that predispose to IFDs. Regarding HBV reactivation, antiviral prophylaxis should be offered in all HBsAg-positive children during therapy with rituximab and for at least 1–1.5 years after the last dose, while monitoring should be continued for at least 1 year post-prophylaxis [3,16]. Additionally, as the recovery of memory B-cells after rituximab may last > 12 months, childhood vaccinations should be reintroduced carefully [23].

### 2.2. Anti-CD33 mAb

Gemtuzumab ozogamicin (GO) is an antibody-drug conjugate approved for CD33+ AML in pediatric patients. CD33 or SIGLEC-3 (sialic acid-binding immunoglobulin-type lectin) is a transmembrane receptor that is expressed ubiquitously in myeloid progenitors, while it is usually found on leukemic myeloid cells (≥80% of AML blasts), and on monocytes, granulocytes, and mast cells, sparing multipotent progenitors and stem cells. Internalization of calicheamicin (covalently attached to GO) induces double-stranded DNA breaks and thereby prompts cell cycle arrest and apoptosis. GO cytotoxicity on immature myeloid cells leads to profound and long-lasting neutropenia, which in turn translates into increased infection risk [6,63,64].

The AAML0531 phase III trial randomized children with de novo AML to standard chemotherapy with or without GO (*n* = 511 in each arm) and calculated no significant increase in infection rates and in events of febrile neutropenia in the GO arm. [48]. However, cumulative treatment-related mortality (TRM) from enrollment through last follow-up without relapse or induction failure tended to be higher in GO recipients (5-year TRM: GO, 8.6% ± 2.5% versus no-GO, 5.9% ± 2.1%; *p* = 0.09). All but one event during intensification occurred before neutrophil recovery and primarily late in the course (mean, 56 days; range, 17 to 93 days) and was infection related.

A former pilot study on GO plus chemotherapy in 340 children with de novo AML reported all-grade infections in 44% to 82% of treatment courses. Grade ≥ 3 febrile neutropenia was documented in 13–31%, while the most common infections were sepsis and catheter-related infections. Median time to neutrophil recovery to an ANC ≥ 1000/μL depended on treatment phase, and was 33 days at the end of induction to 110 days during HSCT [65].

Several studies have used GO monotherapy on a compassionate basis and for bridging to HSCT in children with r/r AML. Grade ≥ 3 febrile neutropenia and infection in these studies occurred at rates of 33.3–69%, while lethal infections arose in 5.9–8.3% of the patients (mainly attributed to sepsis and IFDs—especially aspergillosis—despite prophylaxis) [66,67,68,69,70,71,72]. The NCT02312037 open-label trial on 105 children with r/r AML administered GO monotherapy or GO combined with standard chemotherapy. During induction and consolidation chemotherapy, recorded grade ≥3 infections occurred in 30.2% and 41.5% of the patients, respectively. Although profound neutropenia and lymphodepletion were significantly more prevalent among combination therapy patients, children under GO monotherapy manifested with significantly higher rates of grade ≥3 device-related infection and febrile neutropenia [49].

Clinical trials on children with r/r AML treated with GO combined with cytarabine and another chemotherapeutic (fludarabine or anthracyclines or mitoxantrone or L-asparaginase) demonstrated higher severe infection rates (46.2–55%; ~9% with grade ≥3 catheter-related infections, ~10% with IFDs, and ~10% with viral infections including SARS-CoV-2 and HSV) and rates of febrile neutropenia (28.9–84.6%), but fatal infections did not exceed 4.4% in all cohorts and corresponded primarily to Gram-negative sepsis [73,74,75]. Moreover, GO in combination with busulfan and cyclophosphamide as a myeloablative conditioning regimen prior to allo-HSCT has been associated with bloodstream bacterial infections in 41.7% of patients and sepsis in 25% [76]. A case series of infants with M5 AML revealed an interesting case with post-induction disseminated candidiasis that subsequently underwent two GO courses and allo-HSCT with no active IFD and with 5-day neutropenia as the sole complication [77].

GO supplementary to chemotherapy was also utilized in pediatric r/r AML cases during conditioning and post-HSCT resulting, as expected, in severe infections in ≥50% of patients. GO dose, intensity of regimens, and type of HSCT seem to correlate with infectious complications, but small sample sizes preclude further analysis (*n* = 2–14). Neutropenia is profound and universal in these cases, and beyond expected pathogens (adenovirus, CMV, and HHV6), rare opportunistic infections emerged (such as *Agrobacterium radiobacter*, *Acinetobacter lwoffii*, *Rhodococcus*, *Pantoea agglomerans*, and diphtheroids) [78,79,80,81]. The NOPHO-AML 2004 study randomized 120 children with AML to GO or no further therapy post-consolidation. Neutropenia with ANC <500/μL was almost universal (94–96%) among GO recipients and lasted for a median of 15 days (range: 0–43). Febrile neutropenia treated with antibiotics followed 40% of the GO courses. Children who developed one episode of febrile neutropenia after the first course had a 74% risk of a second episode after the second GO course, compared with only 7% of those without an episode of febrile neutropenia after the first GO course (*p* < 0.001). Nevertheless, none of the infectious episodes were life-threatening [50].

In view of the available data, the impact of GO therapy on infection risk cannot be determined accurately, but phase II and III studies demonstrate rates of infection, such as comparator groups. Prophylaxis against HSV, VZV, CMV, and PJP, along with HBV reactivation prevention, should follow established supportive guidelines for AML. Post-marketing surveillance calculates the severe infection risk at 28.4–32.8% with GO monotherapy (sepsis and bacteremia in one-fourth of cases) and at 42.2–76.3% when GO is combined with chemotherapy and directs clinicians’ attention to IFDs and bacterial infections even from uncommon species, such as *Stenotrophomonas* spp. [6,63]. Apart from prior and concomitant immunosuppressive therapies, premedication with corticosteroids may also play a role but is essential due to the risk of infusion-related reactions with GO [6].

### 2.3. Anti-PD-1 mAb

Pembrolizumab is an immune checkpoint inhibitor directed against PD-1. PD-1 is mainly expressed on activated CD4+ and CD8+ T-cells, but also on B-cells, monocytes, natural killer (NK) cells and dendritic cells (DCs), and can be triggered by two ligands, PD-L1 and PD-L2. Binding of PD-1 to either ligand in the presence of T-cell receptor protein complex (TCR) delivers a co-inhibitory signal that terminates the TCR/CD28 signal and results primarily in a profound inhibition of CD8+ T-cell effector functions, leading to their exhaustion. PD-L1 has been found on the surface of tumor cells and of various cells present in the tumor microenvironment, contributing to escape from immune surveillance. T-cells infiltrating tumor tissues secrete interferon-gamma (IFN-γ), which triggers regulatory immunosuppressive loops, including PD-L1 expression. Therefore, upregulation of PD-1 expression reflects an active T-cell infiltrate, and the intensity of PD-1/PD-L1 staining gives a hint of the expected clinical benefit in many tumors [3,7]. Immune evasion is a common trait of Hodgkin lymphoma (HL), and beyond the aforementioned cytokine-mediated pathway, there are two more characteristics of HL pathogenesis that result in overexpression of PD-L1 and PD-L2: overactive JAK-STAT signaling pathway (Janus kinases and signal transducers and activators of transcription; together with the constitutive NF-κB activation) and amplification or gains of chromosome 9p24.1, where their genetic loci reside [82]. Consequently, PD-1 blockade restores the activity of anti-tumor CD8+ T-cells, enhances NK-mediated ADCC, leads to secretion of cytokines, and attracts APCs [3].

The KEYNOTE-051 phase I/II trial is the only one that has published results on the use of pembrolizumab in children with r/r lymphoma. In terms of treatment-related infectious complications in the whole cohort, one patient had grade 3 neutropenia, one case of infectious enterocolitis was noted, one patient succumbed to sepsis at day 21 following the first pembrolizumab administration (0.6% each), and three patients presented with grade ≥3 lymphopenia (1.9%). The absolute counts of memory B- and T-cells seemed to generally increase after pembrolizumab treatment and minimal differences in antibody titers of tetanus toxoid and *S. pneumoniae* were noted pre- and post-treatment [83]. Final results from the KEYNOTE-051 and results from the KEYNOTE-667, a phase II, open-label study of pembrolizumab in children with de novo HL and slow early response to frontline chemotherapy, are eagerly anticipated [84]. The KEYNOTE-087 and KEYNOTE-204 trials on adults treated with pembrolizumab for r/r HL observed severe neutropenia in only 2–2.4% with limited grade ≥3 infectious complications (4.2%; 0.7% with IFD), concerning mainly the respiratory system [85,86].

Although there is no intrinsic increase in infection rates in children under pembrolizumab, the treatment is not devoid of toxicities, whereas combination with chemotherapy turns severe pneumonia from rare to common [7]. Patients with immune-related adverse events who are expected to receive 20 mg of prednisone daily (or equivalent) for 4 weeks need PJP prophylaxis [87]. There is also a minimal but considerable risk of immune reconstitution inflammatory syndrome (IRIS) in cases that PD-1 blockade unmasks latent *Mycobacterium tuberculosis* infections or chronic progressive pulmonary aspergillosis within 3 months of treatment [88]. Vaccinations should be performed according to standard practice, as there is no indication of memory cells’ impairment [83].

## 3. Bi-Specific T-Cell Engagers (BiTEs)

Blinatumomab is a BiTE specific for CD3 and CD19 that directs CD3-positive cytotoxic T-cells to destroy CD19-expressing B-cells. Transmembrane protein CD19 presents with B-cell lineage commitment and its expression increases with B-cell development, until differentiation into plasma cells, while it is also found in precursor B-cell lymphoblasts. Compared with CD20, it is expressed at earlier developmental stages of B-cells. CD19 is tied with B-cell signaling for survival, proliferation, differentiation, and death. Recruitment of cytoplasmic signaling proteins to the membrane is its major role, while the CD19/CD21 complex is responsible for a threshold decrease in BCR signaling. In addition, anti-CD19 agents (as reported for anti-CD20 agents) seem to affect B-cell-dependent activation of T-cells. Other functions of CD19 implicate complement activation and interaction with APCs by means of major histocompatibility complex (MHC) class II-mediated signaling. Blinatumomab anti-tumor activity is accomplished by the formation of a synapse between the T-cell and the tumor cell independently of MHC, by upregulation of cell adhesion molecules, production of cytolytic proteins, the release of inflammatory cytokines, and proliferation of T-cells, which result in directed CD19+ cell lysis. Consequently, depletion of CD19-positive B-cells has been associated with HGG and delayed recovery of CD4+ T-cells, whereas CD8+ T-cells recover early [3,8,89]. As a precaution against cytokine release syndrome, premedication with prednisone or dexamethasone is routinely administrated, but it does not associate with infections [8].

The RIALTO study on 110 pediatric r/r BCP-ALL cases who received blinatumomab showed a well-tolerated safety profile: justified infections in 45.5% of cases (treatment-related all-grade 8.2% and grade ≥3 3.6%), treatment-related HGG in 4.5%, and febrile neutropenia in 2.7% [90]. Grade ≥3 infections were mainly device-related along with Gram-negative sepsis and IFDs [91]. Former phase I/II trials in pediatric r/r BCP-ALL patients estimated rates of severe infectious complications at 17–22% and HGG at 11%, but all fatal infections (sepsis and IFD) were deemed unrelated to treatment [92,93]. Two published phase III trials in children with first-relapse B-ALL randomized to either blinatumomab or standard intensive chemotherapy prior to HSCT reported lower rates of infections in the blinatumomab arm (Table 2). The first trial found no significant decrease in serious treatment-related infection rates (5.6% vs. 7.8%; described one case of HSV), but HGG was evident only in the blinatumomab arm. The second trial reported significantly lower grade ≥3 infection rates in the blinatumomab group (~10% in each cycle) [51,52].

Retrospective studies of blinatumomab as a bridge to HSCT in children with r/r BCP-ALL revealed higher rates of grade ≥3 infections (18.4–33.3%), mainly IFDs (5.1–10.5%; aspergillosis, PJP, candidiasis, and mucormycosis), presumably due to small sample sizes and preceding chemotherapy regimens. Remarkably, high rates of infection-related deaths in these cohorts (4.6–11.1%) were associated with IFDs and PML [94,95,96,97,98,99]. Similarly, preliminary studies on children with r/r ALL who received blinatumomab under compassionate use post-HSCT showed severe infections in one-third of the cohorts and fatal infections in 22.2%, but these events likely were not treatment-related [100,101].

Two retrospective studies that have investigated the efficacy and safety of compassionate inotuzumab ozogamicin and/or blinatumomab in children with r/r B-ALL showed three infection-related deaths in the first arm and one in the second, but no deaths were thought to be treatment-related [53]. Intriguingly, blinatumomab in the second study offered a non-myelosuppressive alternative for two patients with severe opportunistic infections, i.e., invasive mucormycosis and severe *Pseudomonas* infection, which allowed the delivery of effective antifungal antibiotic and antibiotic therapy without compromising the delivery of effective anti-leukemia therapy [102]. For children with B-ALL and concurrent IFDs, conventional chemotherapy is withheld during antifungal treatment and immune recovery, and substitution with repeated cycles of bridging blinatumomab showed clinical benefit. IFDs following blinatumomab administration in children with r/r ALL are uncommon (<1%), due to the fact that fungal infections are promoted by impairment of phagocytes and T-lymphocytes, but not of B-cells [103]. In addition, blinatumomab showed a clear benefit for pediatric patients who could not tolerate cytotoxic therapy and needed time for immune reconstitution. Beyond the initial drop of lymphocytes on day one of treatment, no other hematologic toxicity was noted, except from one case of *Staphylococcus aureus* bacteremia (9.1%) that was successfully treated with antibiotics [104].

Therapy with blinatumomab is not expected to increase meaningfully the infection risk compared with conventional chemotherapy regimens for r/r ALL. Post-blinatumomab catheter-related infections are associated with the need for a 4-week intravenous infusion and HGG, which should be corrected with administration of IVIG, especially in cases of recurrent infections. Moreover, vaccination with live virus vaccines is not advised for at least 2 weeks prior to the start of treatment, during treatment, and until immune recovery following the last cycle of blinatumomab [16].

## 4. Tyrosine Kinase Inhibitors (TKIs)

### 4.1. Imatinib

Considering the mechanism of action of imatinib as a potent inhibitor of the adenosine triphosphate (ATP)-binding pocket of the BCR-ABL1 fusion protein (and of others mentioned in Table 1), no remarkable increase in infections is anticipated. However, myelotoxicity occurs after long-term use in animal models, along with an increased rate of opportunistic infections. Lymphopenia and neutropenia have been confirmed in humans, especially in blastic-phase CML [9]. In general, imatinib treatment confers only a modest increase in the risk of infection, most likely due to off-target inhibition of kinases involved in the function of immune cells: dose-dependent inhibition of CD4+ and CD8+ T-cell proliferation, interference with T-cell activation by inhibiting LCK, which oligomerizes, binds to, and phosphorylates the TCR, impaired CD8+ T-cell responses against EBV and CMV, inhibition of differentiation and function of CD34+ DCs and CD4 + CD25+ T_reg_ cells, and disruption of B-cell signaling and survival (via dose-dependent inhibition of Bruton’s tyrosine kinase and its downstream substrate phospholipase-C-γ2), resulting in reduced memory B-cells [105,106]. Imatinib treatment in children with chronic phase CML (CML-CP) prompts low-for-age immunoglobulin levels and IgG, IgA, and IgM levels are reduced in 30%, 27%, and 33% of patients, respectively (17% with pan-HGG), while HGG is associated neither with the duration nor with the dose of imatinib treatment [107].

The NCT00845221 phase IV trial conducted in 44 pediatric patients with newly diagnosed CML-CP reported no severe infections, but high rates of neutropenia (all-grade 88.6%; grade ≥3 27.3%) that led to dose reduction or treatment discontinuation in 4.5% of the patients [108]. The CML-PAED-II phase III trial investigated imatinib as frontline treatment in 148 children with CML at all phases and found all-grade infections in 25.7% of cases (mostly upper respiratory tract infections), whereas severe neutropenia was evident in 14.9% of patients. Grade 3 pneumonia was diagnosed in four patients (2.7%), resulting in a temporary interruption of imatinib [109]. Comparable rates of infections (all-grade 16.7% and grade ≥3 infections 3.3%) were reported in an early phase II study on 30 children treated for CML in late chronic, advanced phase, or in relapse after HSCT. The rate of severe neutropenia in this cohort was 16.7% [110]. Another phase II study, AAML0123, evaluated 51 pediatric patients with de novo CML-CP treated with a higher dose of imatinib (340 mg/m^2^). No severe infection was noted, lymphopenia was found in 10% (but predominantly during the first course), and grade ≥3 neutropenia in 32% of cases [111]. The same team published the results of a phase I study (P9973) on 31 children who received imatinib for Ph-positive leukemias and reported three grade ≥3 infections (9.7%), i.e., two catheter-related and one severe infection without neutropenia. The higher rate of infectious complications in this trial can be explained by the inclusion of children with Ph-positive ALL [112].

Higher rates of infection were observed in children with Ph-positive ALL treated with chemotherapy and imatinib, who underwent HSCT (EsPhALL2004, EsPhALL2010, AALL0031, and ALL/SHOP-2005 trials). Infection rates ranged from 16.4% to 58.3% depending on chemotherapy regimen, imatinib dose and phase of treatment, with significantly lower rates in consolidation compared with induction. Grade ≥3 infections were mainly bacterial infections, sepsis, and catheter-related infections, while severe neutropenia was typical for nearly all patients. IFDs occurred in 1.7–5.7% of participants, and in addition, relatively high HSV infection rates (up to 9.1%) were noted. Fatal infections occurred in 3.9–8.4% of these cohorts, including one remarkable case of septic shock due to *Aeromonas hydrophila caviae* after intestinal necrosis [54,55,56,113]. A phase III study compared the effect of imatinib versus dasatinib in addition to intensive chemotherapy treatment for pediatric patients with Ph-positive ALL and found no significant differences in infection rates: 32.6% vs. 30.9% for grade ≥3 infections, 5.3% vs. 5.3% for fatal infections, and 7.4% vs. 7.5% for IFDs, respectively. No fatal event was attributed directly to the TKIs [57].

Retrospective studies on children with CML-CP treated with imatinib report generally low infection risk (≤4%; mainly pneumonia) and low rates of febrile neutropenia (≤3.3%), despite the observed high rates of neutropenia (all-grade 14.9–52.8%; grade ≥3 4.2–9.7%) [114,115,116,117,118,119,120,121,122,123,124,125,126,127]. Only two infection-related deaths were reported in these studies (*n* = 502; 0.4%); one case of tuberculous meningitis in a patient with a complete hematological response, and one case of sepsis in a patient with blast crisis [118].

TKI administration should be preceded by screening for chronic HBV infection and antiviral prophylaxis is mandatory during therapy in HBsAg-positive patients. There is no expected benefit from routine antibacterial, antiviral, or anti-PJP prophylaxis, with the exception of cases that need individualized infection risk assessment due to extensive immunosuppression [105]. Notably, CML patients on TKIs exhibit significant impairment of humoral IgM responses to pneumococcal vaccines compared with controls, which is likely due to a significant loss of peripheral blood IgM memory B-cells, whereas immune responses to influenza vaccination are comparable with controls [106].

### 4.2. Dasatinib

Similar to imatinib, dasatinib confers myelotoxicity and, apart from the aforementioned impact of TKIs on the immune system, dasatinib has been associated with inhibition of CD8+ T-cell proliferation (especially of CMV- and influenza matrix proteins-specific CD8+ T-cell responses) [105]. Dasatinib is a second-generation TKI with 325-fold higher potency than imatinib against ABL kinase that displays affinity to multiple sites within the kinase and has the advantage of crossing the blood-brain barrier. Dasatinib seems to overcome imatinib resistance originating from BCR-ABL1 point mutations (with the exception of T315I), activation of alternate signaling pathways (such as Src kinases), and multi-drug resistance gene overexpression [10].

Dasatinib confers a slightly higher infection risk than imatinib in children suffering from Ph-positive ALL, CML in any phase other than CP, and after HSCT [10]. The AALL0622 phase II/III trial on 59 pediatric patients with Ph-positive ALL found the combination of dasatinib plus intensive chemotherapy to be safe and feasible. Grade ≥ 3 infections were diagnosed in 15.3%, 72.9%, 67.8%, and 68.4% of cases during the induction, post-induction, intensive, and maintenance phases of treatment, respectively (sepsis 0–5.3%; colon infections 0–15.3%) [128]. A phase III study of dasatinib versus imatinib added to intensive chemotherapy in children with Ph-positive ALL found comparable rates of infectious complications: 30.9% with grade ≥3 infections, 7.5% with IFDs, and 5.3% with fatal infections (no treatment-related case) in the dasatinib arm [57]. The CA180-372 phase II trial that utilized dasatinib in combination with chemotherapy in 81 children with Ph-positive ALL found high grade ≥3 infection rates: 86% febrile neutropenia, 31% sepsis, 25% pneumonia, 20% bacteremia, 14% *Clostridium* infection and urinary tract infection, 12% viral infections, 11% IFDs, 10% upper respiratory tract infections and sinusitis, and 3.7% fatal infections, none of which was dasatinib-related [10,129,130]. The CA180-018 phase I study investigated 58 children with r/r leukemia receiving dasatinib and found severe neutropenia in 59.6% of cases, affecting typically cases of Ph-positive ALL and advanced CML and not CML-CP patients [129]. The CA180-226 phase II trial on 113 pediatric patients treated with dasatinib for CML-CP cited only one case of a grade ≥3 infection in a patient that had been previously treated with imatinib (0.9%; no incident in the de novo group) [130]. In the latter trial, one case relapsed while on dasatinib and subsequently developed fatal cerebral aspergillosis [131]. An open-label study that used dasatinib combined with standard chemotherapy in 20 children with core-binding factor (CBF) AML observed no infections and low rates of severe neutropenia (14.3%) [132].

A retrospective study of 450 patients with CML-CP treated with TKIs for over 1 year revealed no meaningful risk of severe infection among three TKIs (0.4% for imatinib; 1.1% for dasatinib; 0% for nilotinib) [133]. Sequential use of second-generation TKIs in children with CML-CP following imatinib therapy was associated with no infections, while severe neutropenia was found in 6.7% of the dasatinib-treated patients and in none of the nilotinib group [134]. In a single child treated with dasatinib for Ph-positive ALL, whole blood functional assays displayed abolished CMV-specific T-cell responses. In this case, refractory leukemia under dasatinib was followed by CMV, EBV and adenovirus infection, despite the presence of lymphocytosis with a high CD8+ T-cell count. Intriguingly, cessation of dasatinib lead to prompt clearance of all three viruses. This type of immunosuppression is likely to result from the blockade of TCR triggering via inhibition of LCK phosphorylation [135]. Of interest, opportunistic infections (IFDs, CMV, tuberculosis, VZV, and reactivation of HBV) have been described with dasatinib use, particularly among HSCT recipients. Screening for chronic HBV infection should be routinely performed before TKI initiation and prophylaxis should be advised only after an individualized infection risk assessment [105,136]. Adhering to the guidelines of the 2017 European Conference on Infections in Leukaemia (ECIL 7) for children with hematological malignancies, TKIs are accounted as only mildly immunosuppressives, and all attenuated vaccines (including influenza and pneumococcal conjugate vaccine) can be given during treatment, though their efficacy may be lower (75–85%). Although the data are scarce, a window for live attenuated vaccination can be created in children with CML receiving TKIs by temporarily interrupting the therapy. Despite the concerns for the safety of vaccines that contain live attenuated viruses, preliminary experience supports the safety of MMR vaccine administration, while indications of COVID-19 vaccination for children with CML do not differ from those for the general pediatric population [137].

### 4.3. Nilotinib

Safety data from nilotinib use in children are limited. The CAMN107A2120 phase I study included 15 children with Ph-positive CML or ALL treated with nilotinib and found no significant risk of infection compared with adult trials: all-grade nasopharyngitis in 26.7% (all <10 years old), mild fungal infections in 13.3% (skin infection and oral candidiasis), and catheter-related infection in 6.7%. Respiratory tract infections were common, but not serious, and profound neutropenia was found in 13.3% of patients (all ≥10 years old) [138]. The CAMN107A2203 phase II study investigated the use of nilotinib in 58 children with Ph-positive CML-CP and found upper respiratory infections in 31% of cases (1.7% grade ≥3) and nasopharyngitis in 19% (none grade ≥3) [139]. Moreover, a long-term update of this trial observed no new infection-related safety signals with prolonged treatment [140].

Sequential use of second-generation TKIs following treatment with imatinib in children with CML displays zero risk for severe neutropenia in patients receiving nilotinib [134]. Strikingly, pediatric patients with CML-CP are usually found with concurrent respiratory infection at diagnosis, while infectious adverse events are expected in 14% of cases irrespective of the therapy regimen followed [141]. In this context, nilotinib use is marked as safe in terms of infection risk. Safety data from adults report a risk of <1% for infection with nilotinib use in CML-CP (most cases with respiratory tract infections) [11]. Recommendations for screening, prophylaxis, and immunizations are the same with imatinib and dasatinib [105].

### 4.4. Crizotinib

Safety data are still limited for crizotinib use in the pediatric population. The possible need for long-term or even life-long therapy raises questions about the totally uncharted long-term toxicity and if resistance will eventually develop [142].

The ADVL0912 phase I study included nine children with anaplastic large cell lymphoma (ALCL) treated with crizotinib and found grade ≥3 lymphopenia and neutropenia in 8.3% and 12.5% of cases after the first cycle of treatment, respectively. Grade ≥ 3 infections were justified in one case after the first cycle and in four cases in subsequent cycles (involving catheter-related infections, disseminated VZV, and viral gastroenteritis) [143]. The ADVL0912 phase I/II study on 26 children receiving crizotinib for ALK-positive ALCL noted treatment-related grade ≥3 neutropenia (61.5%), lymphopenia, febrile neutropenia, myositis, and skin infection cases (3.8% each; no severe infection in the low-dose group) [144]. A commentary on ADVL0912 stated that rates of severe cytopenias may rise with exposure and that upper respiratory infections are commonly seen with crizotinib treatment (31%; no severe case) [145]. Combination of crizotinib with cytotoxic chemotherapy in the ADVL1212 phase I study for children with refractory solid tumors or ALCL demonstrated high rates of severe infections (20.5%) and febrile neutropenia (38.5%) [146]. The ANHL12P1 phase II study randomized children with ALK-positive ALCL to either crizotinib or brentuximab vedotin combined with chemotherapy and found no significant difference in infection rates, even though serious adverse events were more likely to occur in the crizotinib arm (OR 2.89; 95% CI: 0.73 to 11.4; *p* = 0.06). Serious adverse events and other infectious complications were noted in 11.8% and 25% of children under crizotinib, respectively [58].

### 4.5. Entrectinib

Safety data on children receiving entrectinib (RXDX-101) are lacking, but based upon its mechanism of action, it is unlikely to confer immunosuppression. Its use in pediatric hematological cancers is still discussed, regardless of its tumor-agnostic action. The STARTRK-NG phase I/II study on pediatric patients (none with NHL) treated with entrectinib for locally advanced or metastatic solid tumors or primary CNS tumors and/or who had no satisfactory treatment options, revealed no increased risk of infection (grade ≥3 infections, neutropenia, and lymphopenia in 2.3%, 7%, and 2.3%, respectively; mainly lung and urinary tract infections; significantly more device-related infections than in adults) [13,147]. Logistic regression models indicated that the probability of a patient experiencing a grade ≥3 treatment-related or a serious adverse event increased with entrectinib exposure > 600 mg/day [148].

### 4.6. Larotrectinib

Similar to entrectinib, larotrectinib has not been established in treating hematological cancers, but pan-Trk inhibitors are not expected to predispose to infections. A recent study showed that oncogenic *NTRK* fusions are amenable to inhibition in hematologic malignancies, although they correspond to only 0.1% of cases [149]. *NTRK* fusions were identified in 2.2% of pediatric tumors (including T-lymphoblastic lymphoma), but larotrectinib use in pediatric hematologic cancers remains on a theoretical base. [150]. Data from the three published phase I/II trials on children treated with larotrectinib for *NTRK* fusion-positive solid tumors observed grade ≥3 infections in up to 27.3% (lung and urinary tract infections, influenza, and viral gastroenteritis; none were considered treatment-related) and profound neutropenia in 4.2–28.8% of patients [151,152,153].

## 5. Chimeric Antigen Receptor T-Cells (CAR T-Cells)

### Tisagenlecleucel

Tisagenlecleucel recipients are constitutively at risk for infections, mainly due to prolonged leukopenia, depleted B-cells, and low immunoglobulin levels. Concurrent predisposition factors to severe infections are prior chemotherapy regimens (>3), high doses of CAR T-cells (2 × 10^7^ cells/Kg), and occurrence of the cytokine release syndrome (CRS) or immune effector cell-associated neurotoxicity syndrome (ICANS; previously referred to as CAR-T-cell-related encephalopathy syndrome or CRES). Both latter clinical entities require the administration of tocilizumab as an anti-inflammatory agent (an anti-IL-6 mAb) and dexamethasone, respectively, which both increase the infection risk. IFD occurrence has been associated with HSCT and CRS, while 1% of patients are expected to develop IFDs under prophylaxis (≤14.3% without). The recommendations for antifungal prophylaxis include prior mold infection, ≥3 weeks of neutropenia before and after infusion, and treatment with dexamethasone >0.1 mg/kg/day for at least one week [3,4]. In children who have previously been subjected to HSCT, the recommendation is to monitor CMV, adenovirus, EBV, and HHV6 once or twice a week. Routine prophylaxis for bacterial infections is not indicated, acyclovir is recommended for HSV seropositive children, and PJP prophylaxis is recommended for all cases [15,154]. The European Society for Blood and Marrow Transplantation recommends the administration of granulocyte colony-stimulating factor to shorten the duration of neutropenia 14 days post-infusion [155].

The ELIANA phase II study investigated 75 pediatric patients with r/r CD19-positive ALL who received tisagenlecleucel therapy. The probability of maintenance of B-cell aplasia at 6 months after the infusion was 83% and treatment-related grade ≥3 neutropenia was apparent in 9.3% of patients during the first two months post-infusion (2.9% at ≥8 weeks post-infusion). Grade ≥3 febrile neutropenia was noted in 34.7% of patients, while infections were seen in 42.7% of children (24% grade ≥3: upper respiratory tract infections and RSV, sepsis, bacteremia, and encephalitis). Two infection-related deaths were recorded (2.7%; both >30 days after infusion): one due to HHV6 encephalitis and one due to an IFD [156]. One more fatal bacterial lung infection was reported long after infusion in a safety update of ELIANA, and was attributed to the subsequent therapies [157]. Remarkably, a safety report regarding the six Japanese patients of ELIANA phase II study noted one case of grade 3 pneumonia, one case of grade 3 upper respiratory tract infection, one case of grade 3 bacterial meningitis, and one case of grade 4 viral encephalitis (16.7% each; serious infections in two-thirds of patients) [158]. A phase I study on CAR T-cell therapy involving 25 pediatric patients with r/r B-ALL found infections in 36% of cases (grade ≥3 were 24%) and profound neutropenia in 12% [159]. A larger phase I/II study on 45 pediatric patients with r/r CD19-positive ALL undergoing CAR T-cell therapy estimated the rate of grade ≥3 febrile neutropenia at 4.7% of cases, but no severe infection was cited [160]. Another phase I study on 21 pediatric patients with r/r ALL or NHL noted grade ≥3 febrile neutropenia in 36.8% of cases, and the median duration of neutrophil count <500/μL was 8 days (0–38 days in responding patients), but prolonged (≥14 days) grade 4 neutropenia was noted in one-third of patients [161]. Pooled safety analysis of 137 pediatric patients from ELIANA and ENSIGN trials revealed three infection-related deaths (2.2%; due to systemic candidiasis, HHV6 encephalitis, and lower respiratory tract infection). In the latter study, grade ≥3 febrile neutropenia was present in 33.6% of cases, and all-grade infections were justified in 42.3% (19% were grade ≥3). Viral infections were diagnosed in 13.9% (4.4% were grade ≥3; five with rhinovirus, two with HSV1, two with HHV6, two with viral encephalitis, and two with norovirus gastroenteritis), bacterial infections in 17.5% (9.5% were grade ≥3; *Staphylococcus* and *Clostridium difficile* infections), fungal infections in 5.8% (2.2% with IFD; mainly due to *Candida*), and infections due to unspecified pathogen in 21.2% of cases. Infections developed in 35.8% of patients ≤4 weeks post-infusion (14% were grade ≥3) and in 14.3% of patients 4–8 weeks post-infusion (8% were grade ≥3). Moreover, 34.6% of patients with prolonged grade ≥3 neutropenia developed grade ≥3 infections after day 28 [162]. Another study that reviewed ELIANA, ENSIGN, and B2101J phase II trials for tisagenlecleucel in treating pediatric patients with r/r B-ALL estimated all-grade infections and HGG rates at 65% and 47%, respectively [163].

Prospective and retrospective studies on pediatric patients under tisagenlecleucel reported severe infections in 29–46.3%, corresponding largely to bacterial infections, while IFDs occurred in ≤3% of treated patients. Clinically significant infections were far more frequent early post-infusion (73.7% at days 0–28), and fatal infections pertained to 3.1–5% of cohorts. Contradictory results have been published regarding the duration of B-cell aplasia. The prevailing theory suggests that it lasts 3–6 months in half of the patients and more than 12 months in the remaining half. Grade ≥ 3 neutropenia is observed in <40% of patients and the cumulative incidence of neutrophil recovery at 28 days was 75%. HGG was pronounced in roughly half of the cohorts, while irAEs, pre-therapy lymphopenia, and duration of low IgG were significantly associated with infection density [164,165,166,167,168,169,170,171]. According to the FDA Adverse Event Reporting System for tisagenlecleucel in the post-marketing period for the pediatric population, infection was the second most frequent cause of death (11.3%) after disease progression [172]. Another post-marketing surveillance study of tisagenlecleucel in adults and children revealed a relatively small number of children undergoing CAR T-cell therapy (<0.5%) and higher fungal infection rates (5.4%) [173]. Not surprisingly, treatment of r/r ALL pediatric patients with other types of CD19-specific CAR T-cells documented comparable with tisagenlecleucel grade ≥3 infection rates (21–24%) [159,174].

Prospective data on immunoglobulin replacement after therapy with tisagenlecleucel may be currently scarce, but severe HGG increases the risk for respiratory tract infections and infections from encapsulated bacteria (e.g., *Streptococcus pneumoniae* and *Haemophilus influenzae* type b). Monitoring of serum IgG levels is advised along with the administration of IVIG ~1 month after the infusion of tisagenlecleucel (0.5 g/kg/monthly) to keep the serum levels of IgG close to the normal range for the child’s age. IgG levels >800 mg/dL are recommended especially for children <10 years of age and when there are relevant risk factors (baseline pulmonary pathology, history of total body irradiation, or added immunosuppression by chronic graft-versus-host disease). Replacement therapy should last as long as B-cell aplasia and HGG persist [154,175].

## 6. Other Targeted Therapies with Off-Label Use

Table 3 describes the most common targeted therapies that have not yet gained FDA or EMA approval for treating pediatric patients with hematological malignancies.

### 6.1. Inotuzumab Ozogamicin

Inotuzumab ozogamicin (InO) is an antibody–drug conjugate (ADC) mAb that is a potential future milestone for second-line treatment of r/r BCP-ALL in association with blinatumomab [2]. CD22 or SIGLEC-2 is a sugar-binding transmembrane protein predominantly found on the surface of mature B-cells and on up to 90% of B-cell blasts, and to a lesser extent on some immature B-cells. CD22 seems to regulate BCR activation and subsequently plays a role in B-cell activation and survival, while it also serves as an adhesion molecule [3].

Phase I/II studies (including AALL1621, ITCC-059, and INO-Ped-ALL-1) displayed a well-tolerated safety profile for children treated with InO for r/r CD22-positive ALL. Grade ≥3 infections were microbiologically confirmed in 10.5–17.6% of patients and were predominantly pneumonia and sepsis, followed by catheter-related and skin infections, occurring within two months, and considered as unrelated to treatment in their vast majority. Grade ≥3 febrile neutropenia was recorded in 21.4–32% of the patients and neutropenia was ubiquitous (92.9%; 18.8–56% grade ≥3). Likewise, B-cell depletion was universal, but there was no significant decrease in peripheral T-cells post-InO regardless of response [179,180,181,182,183].

Promising results of InO in children with r/r ALL were reported in 2013, even though this initial publication mentioned one fatal case of sepsis among five children [184]. Retrospective studies in children with r/r B-ALL under compassionate administration of InO documented infections in 29.4–50% of patients [19.6% grade ≥3: sepsis, bacteremia, IFDs (3.9%), gastrointestinal, and respiratory tract infections]. Grade ≥3 neutropenia was estimated at 83%, grade ≥3 febrile neutropenia at 11.8%, B-cell depletion lasted from day 8 of the first course to day 16 after the last course, while T-cells remained >150/μL in all studied patients, allowing subsequent CAR T-cell therapy [185,186].

According to the InO mechanism of action, it is assumed that the risk of infection will not increase meaningfully. Almost all patients under InO are expected to experience B-cell depletion within 4 weeks of therapy initiation, and B-cell recovery will occur at 9–12 months post-InO. Serum immunoglobulin levels are not expected to fluctuate (expect from a slight decrease in Igs). Prophylaxis is not advised, but infection risk with InO should be individually evaluated, along with parameters of the underlying disease, comorbidities, and concomitant immunosuppression [3,63].

### 6.2. Brentuximab Vedotin

Brentuximab vedotin (BV) has been successfully administered in r/r pediatric lymphomas during recent years. The corresponding SPC includes a boxed warning for PML along with precautions for the emergence of severe and opportunistic infections. BV is directed against CD30, a member of the TNF receptor family -rarely found on healthy tissues- that resides on the surface of ALCL and Reed-Sternberg cells (but variably expressed in other T-cell lymphomas). The BV mechanism of action relies on the disruption of CD30-CD30 L signaling (blocking signals of survival and proliferation) and the induction of apoptosis and ADCP by internalization of the BV-CD30 complex and the release of tubulin-disrupting monomethyl auristatin E [178]. However, CD30 can be found also on activated T-, B-, NK-cells, and monocytes, thereby affecting the balance between Th1 and Th2 responses and the generation of memory and effector T-cells. In the latter case, CD30 blockade exerts a deleterious effect on humoral immunity and ADCC processes [63].

The COG ANHL12P1 trial on 68 children with newly diagnosed ALCL added BV to the ALCL99 regimen and reported grade ≥3 infections, oral mucositis, febrile neutropenia, and neutropenia in 4.5%, 5.5%, 13.5%, and 24.1%, respectively. Severe infections in this trial included lung infections, urinary tract infections, catheter-related infections, sepsis, skin infections, and appendicitis [60]. Interestingly, no difference in infection rates has been calculated compared with the historical ALCL99 cohort [61]. A phase I/II study involving 36 children with r/r classical HL or ALCL under monotherapy with BV published low rates of all-grade treatment-emergent infectious adverse events [187]. A similar but smaller, phase I study on six children reported neutropenia in 83.3% (33.3% was severe) and grade 2 upper respiratory and skin infections in 50% and 16.7%, respectively [188]. The HLHR13 phase II study on 16 children with de novo high-risk classical HL treated with BV found no difference in grade ≥3 neutropenia and stomatitis compared with the historical GPOH-HD-2002 cohort (81.3% vs. 81.5%, and 6.3% vs. 8.7%, respectively) [59].

BV has been used as consolidation therapy after autologous HSCT in 6 children with early-relapsed HL and one-third of patients developed severe neutropenia, while no case of severe infection was noted [189]. In the same setting, a case series of 5 pediatric patients reported no severe infections, too [190].

A combination of BV with bendamustine in 29 children with r/r HL exhibited a manageable safety profile with 44.8% of patients experiencing severe neutropenia and one episode of grade 3 sepsis (3.4%) [191]. Another study with 29 pediatric patients who received the previous combination found severe neutropenia in 31% of the cohort, while there was a single grade 3 lung infection that resulted in treatment discontinuation [192]. A third study on 32 pediatric patients under BV and bendamustine for r/r HL noted severe neutropenia in 71.9% of patients and no severe infection [193]. The AHOD1221 phase I/II study that combined BV with gemcitabine in 45 pediatric patients with r/r HL displayed grade ≥3 neutropenia in 88.9% of patients and grade ≥3 infections in 15.6% [194]. Not surprisingly, a recent phase II trial on 30 pediatric patients who received BV, rituximab, and risk-adapted chemotherapy for advanced stage, intermediate- and high-risk newly diagnosed HL, displayed lower rates of serious infections with grade 3 mucositis in 3.3% [195]. A trial that used BV instead of vincristine in the OEPA/COPDAC regimen investigated the safety of BV in 77 children with high-risk HL, and denoted low rates of grade ≥3 infections: 13%, 9.1%, 0%, 5.3%, 1.3%, and 0% in cycles 1, 2, 3, 4, 5, and 6, respectively [196].

A retrospective study that involved many pediatric patients with r/r HL cited one case of IFD with BV monotherapy [197]. Pulmonary toxicity seems to occur more frequently among children with r/r HL after BV administration, according to a retrospective study, but results did not reach statistical significance (for pulmonary infection: OR 4; 95% CI: 0.55 to 29.17; *p* = 0.08) [198]. A larger retrospective study of 68 children with r/r HL treated with BV as a single agent or combined with chemotherapy, calculated the occurrence of grade ≥3 neutropenia at 25% and all-grade infections at 22.1% (grade ≥3 at 10.3%; predominantly pneumonia followed by CMV and VZV) [199]. In general, the use of BV was associated with upper respiratory infections (very common) along with sepsis and urinary tract infections (common) [178,195].

Based on available data, therapy with BV does not seem to increase the risk of overall infections meaningfully, given the intrinsically high-risk nature of infections of those with underlying r/r lymphoma. However, treatment with BV bears an additional risk of neutropenia. Anti-HSV and anti-PJP prophylaxis should be routinely administered to autologous HSCT recipients treated with BV, neurological symptoms should prompt investigations for PML, while secondary prophylaxis against CMV is advised for seropositive patients scheduled to receive additional BV doses [63]. The relationship of BV with tuberculosis is under investigation [194].

### 6.3. Bortezomib

The boron atom in bortezomib inhibits with high affinity and specificity—but reversibly—the catalytic site of the 26S proteasome that normally exhibits chymotrypsin-like activity by degrading ubiquitinated proteins. The disruption of the ubiquitin–proteasome pathway leads to loss of cell homeostasis and thereby induces cell-cycle arrest and apoptosis [200]. T-cell depletion is commonly seen with bortezomib treatment, thus impairing viral antigen presentation, and resulting in a higher risk for reactivation of viral infections, particularly of VZV. Of course, the combination of bortezomib with other immunosuppressive regimens raises the risk for opportunistic infections [88].

The COG AALL1231 phase III trial investigated the use of bortezomib in 824 pediatric patients with newly diagnosed T-cell ALL and lymphoma and reported in the bortezomib arm rates of grade ≥3 microbiologically confirmed infections at 2.7%, 5.1%, and 6.7% during induction, consolidation, and delayed intensification, respectively [62]. In the bortezomib arm, serious infections were diagnosed in 19.2% of T-ALL patients (mainly due to sepsis 8.5%; followed by respiratory tract infections) and in 33.7% of T-cell lymphoma patients (11.9% was sepsis; followed by respiratory tract infections). Beyond serious infections, other infections were justified in 25.1% of T-ALL and 30.7% of T-cell lymphoma patients. Whilst infection rates in the historical AALL0434 trial that used no bortezomib had been significantly higher, death due to infection in remission was significantly higher in the AALL1231 cohort [1.3% vs. 0.4%; OR 3.09 (95% CI: 1.24 to 7.72; *p* = 0.008)] [62,201]. The COG AALL07P1 phase II trial evaluated reinduction chemotherapy plus bortezomib in 146 pediatric patients with high-risk ALL in first relapse and reported grade ≥3 sepsis, catheter-related infections, febrile neutropenia, and other infections in 15%, 10%, 25–30%, and 21–29% of patients, respectively. Out of 43 microbiologically confirmed infectious episodes, 83.7% were bacterial (mostly from Gram-positive cocci, followed by Gram-negative rods), 11.6% IFDs (mainly aspergillosis), and 4.7% were viral (HSV). The high incidence of IFDs emphasizes the issue of prophylaxis [202]. The corresponding phase I study that evaluated bortezomib as a single agent in 12 children with refractory leukemias mentioned one dose-limiting case of grade 4 febrile neutropenia (8.3%) [203]. The TACL phase II study on 22 pediatric patients treated with bortezomib plus chemotherapy for relapsed ALL justified grade ≥3 infections in 45.5% of the cohort, but unacceptably high infectious mortality led to chemotherapy alterations along with antibiotic and antifungal prophylaxis (vancomycin, levofloxacin, and voriconazole) in the remaining 6 patients, and no further severe infections were noticed. Three children succumbed to sepsis (13.6%) and one child developed an invasive *Mucor* infection of the sinuses, skin, and lung [204]. In the same context, the corresponding phase I study reported a lethal case of diffuse zygomycosis on day 17 and three cases of grade ≥3 bacteremia (4/10 in total), while recovery of neutrophils was observed at a median of 41 days (range: 28–43) [205]. Another phase I/II study on 37 children with r/r ALL following the TACL scheme showed that 16.2% of patients experienced grade ≥3 infections between days 15 and 22 of treatment. Three patients in this cohort died of IFDs (8.1%), while three more children survived from sepsis [206]. In a phase II study of a modestly intensive reinduction regimen combined with bortezomib in 29 r/r ALL children, the grade ≥3 infection rate was 41.4%, including one case of aspergillosis and actinomycosis [207]. Inconsistently, a phase I study that recruited three children with r/r ALL reported no severe infections, even though severe neutropenia occurred in all patients, and one case developed grade 3 febrile neutropenia following treatment with bortezomib-containing chemotherapy [208].

Data on bortezomib therapy for pediatric AML and HL are limited. The use of bortezomib combined with either idarubicin/cytarabine or cytarabine/etoposide was investigated in 46 children with r/r or secondary AML and found grade ≥3 infections in 37.5% and 47.6% of courses in the first and the second arm, respectively. Three fatal infections were noted in this cohort (6.5%; one due to an IFD despite prophylaxis). Noteworthy, antifungal prophylaxis was required from day 10 of cycle 1, while azole treatment was discouraged from days −2 to +10 due to interactions of bortezomib with the p450 system [209]. The subsequent AAML1031 phase III trial randomized 1097 pediatric patients with de novo AML to standard treatment with or without bortezomib and observed no differences in infectious complications between treatment arms. Microbiologically documented sterile site infections were documented in 8.1% vs. 8.3%, 15.6% vs. 17.2%, 24.1% vs. 27.4%, and 38.1% vs. 34.4% of patients treated without or with bortezomib during induction phases 1 and 2, and during intensification 1 and 2, respectively. *Viridans* group *Streptococci* were the most prevalent infectious agents, while fungal infections occurred in 0.4–1.7% of cases across all phases [210]. The AHOD0521 phase II study of bortezomib in combination with ifosfamide/vinorelbine in 25 pediatric patients with r/r HL observed grade ≥3 infections in 11.7% of treatment courses (5.2% with VZV; 3.9% with bacteremia due to coagulase-negative *Staphylococci* and *E. coli*; one case of urinary tract and one case of soft tissue infection) and severe neutropenia in 52.2% of patients [211]. In addition, peri-transplant bortezomib was used as part of a GVHD prophylaxis regimen in 46 children with leukemia and 5 early fatal infections occurred (<100 days; 10.9%; mostly due to adenovirus) [212].

A retrospective study of six children with refractory ALL treated with bortezomib combined with standard induction chemotherapy revealed severe and prolonged (22–35 days) neutropenia in all patients and grade ≥3 infections in half of them [213]. High rates of grade ≥3 infections (90.9%) were also noted in a retrospective study of 11 children with relapsed ALL [214].

Utilizing published results from the NCT00931918 trial, we calculated a pooled OR of 4.78 (95% CI: 1.32 to 17.32; *p* < 0.01) for VZV infection with bortezomib use [215]. In this context, antiviral prophylaxis with acyclovir or valacyclovir is strongly recommended for VZV-seropositive patients starting from induction and continuing to ≥4 weeks after its cessation. Seronegative patients without a history of primary varicella infection are eligible for the live attenuated varicella vaccine that should be given ≥1 month prior to bortezomib. therapy. With respect to pulmonary infectious complications, influenza vaccine is advised (≥2 weeks before treatment and annually thereafter), while pneumococcal vaccination should be ideally completed at least one month prior to bortezomib administration (PCV13, followed by PPV23 two months later) [88].

### 6.4. Venetoclax

Venetoclax is a treatment option in pediatric r/r AML and ALL, especially when specific genomic profiles are present (e.g., hypodiploid, *KMT2A*-, and *TCF3*-rearranged ALL) [2]. Selective BCL2 inhibition mediated by venetoclax restores the process of apoptosis by displacing pro-apoptotic proteins (like BIM) and by triggering programmed cell death via mitochondrial outer membrane permeabilization and caspases. Additionally, venetoclax demonstrates cytotoxic activity towards tumor cells that overexpress BCL2, while it seems to restore sensitivity to chemotherapy agents [177]. Beyond their role as apoptosis regulators, BCL2 antagonists seem to selectively eliminate plasmacytoid dendritic cells (with implications in antigen presentation) and to reduce IFN-α production [105].

A phase I study on 29 pediatric patients with r/r AML and ALL treated with venetoclax plus chemotherapy reported no significant increase in infection rates, even though febrile neutropenia was documented in 45% of patients [216]. A retrospective study of 18 pediatric patients who received venetoclax for ALL and T-lymphoblastic lymphoma observed grade ≥3 infectious complications in half of the patients: 27.8% were found with sepsis, 27.8% with febrile neutropenia, 5.6% with pneumonia, 5.6% with mucosal infection, and 55.6% with neutropenia [217]. The same research group conducted a study involving 36 pediatric patients with r/r AML under venetoclax combined with chemotherapy and calculated a rate of grade ≥3 febrile neutropenia at 58.3% [218]. A phase I trial that includes 38 pediatric patients with r/r AML from the latter two studies estimated grade ≥3 bloodstream infections, IFDs, and febrile neutropenia in 15.8%, 15.8%, and 65.8% of patients, respectively, while both deaths in the trial were attributed to sepsis (5.3%) [219]. Another recent trial on 25 pediatric patients who received venetoclax plus chemotherapy for r/r ALL harboring heterogeneous genomic profiles displayed rates of grade ≥3 febrile neutropenia at 52% [220]. A retrospective study of eight children who received venetoclax and chemotherapy for AML or myelodysplastic syndrome revealed severe neutropenia in all treatment cycles and grade ≥3 sepsis, cellulitis, and febrile neutropenia in 11.5%, 3.8%, and 23.1% of courses, respectively [221]. Moreover, a phase I study of venetoclax along with low-dose navitoclax and chemotherapy that included 12 children with r/r ALL documented treatment-emergent grade ≥3 sepsis, pneumonia, and bacteremia in 19.1%, 14.9%, and 6.4% of patients, respectively [222]. Of interest, cytochrome P450 is crucial for the metabolism of venetoclax, bortezomib, imatinib, dasatinib, crizotinib, entrectinib, larotrectinib, and MMAE in BV, and their concomitant use with potent CYP3A4 inhibitors (including, but not limited to, azoles, ritonavir, and clarithromycin) should prompt meticulous monitoring for cytopenias and other signs of toxicity, and, ultimately, dose adjustments [9,10,11,12,13,177,178,200].

Given the limited exposure to the drug so far, clinicians should be alert for rare infections. In view of the available data, venetoclax does not definitely affect the risk of infections, and thus extra prophylaxis is currently off the table [105].

## 7. Conclusions

**Figure 1:**
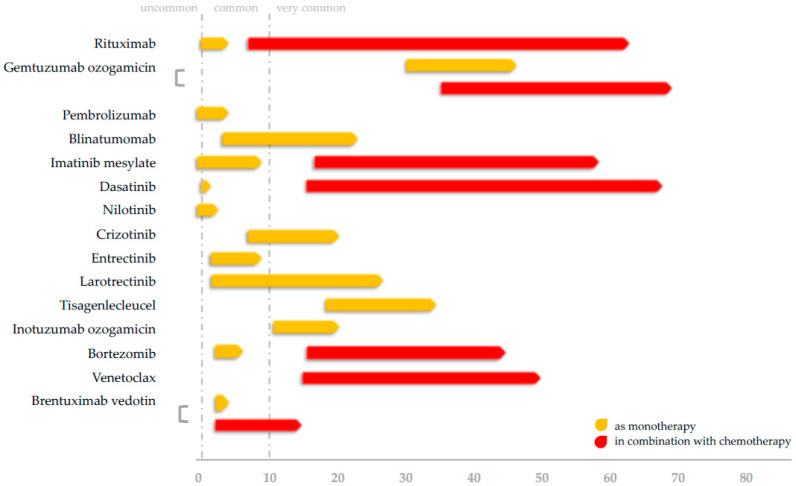
Rates of severe (grade ≥3) infections in children with leukemias and lymphomas administered with targeted agents.

Targeted therapies in children with hematological malignancies are associated with comparable incidence rates to adults (Supplementary Table S1). Higher rates than adults were observed only in agents that have still limited use in the pediatric setting. The exact impact of these agents, which have different mechanisms of action, and are used after conventional chemotherapy or HSCT, is difficult to ascertain. We believe that agents that target a specific immunological pathway seem to be associated with more severe bacterial and fungal infections, as well as with emerging viral infections. Antimicrobial, antiviral, and antifungal prophylaxis should be considered in those patients with severe immunosuppression and with a relapsed or refractory leukemia/lymphoma that are usually treated with more intensive chemotherapy protocols. Table 2 raises the question if infectious complications pose a threat to the outcomes in targeted therapies. Six RCTs have shown significantly different infection rates between children treated with conventional chemotherapy regimens and pediatric patients supplemented with targeted agents [29,52,55,56,62,215]. Noteworthily, all these studies reported higher survival rates for children administered with targeted therapies, so differences in infection rates do not seem to compromise the effectiveness of treatment. In view of infections rates, Figure 1 illustrates a summary of the corresponding (grade ≥3) rates as they have been reported in the literature. In Table 4 we summarize our current knowledge on the risk of specific infectious complications in children with leukemias and lymphomas treated with targeted therapies. Finally, taking into consideration all the above-mentioned data, clinicians should be cautious of severe infections after the use of targeted therapies, especially when used in combination with chemotherapy.

## Figures and Tables

**Table 1:** FDA and EMA approved targeted therapies for childhood leukemias and lymphomas.

Name	Trade Name	Target	Indication	Grade ≥ 3 Infection Risk
**Monoclonal Antibodies (mAbs)**				
Rituximab [5]	MabThera, Truxima, Rituxan, Rixathon, Riximyo, Reditux, Ruxience, Zytux	Chimeric IgG1κ mAb → CD20	In combination with chemotherapy for children ≥ 6 months old with previously untreated and advanced CD20-positive DLBCL, BL, B-AL, or BLL	2.9–4.2% as monotherapy; 7–64% in combination with chemotherapy
Gemtuzumab ozogamicin [6]	Mylotarg	ADC recombinant humanized IgG4κ mAb linked to N-acetyl-gamma-calicheamicin → CD33	In combination with chemotherapy for children ≥ 1 month old with de novo or in children ≥ 2 years old with r/r CD33-positive AML.	30.2–47% as monotherapy; 35.6–69.1% in combination with chemotherapy
Pembrolizumab [7]	Keytruda	Humanized IgG4κ mAb → PD-1	For children with r/r HL and NHL (PMBCL) after ≥ 3 and ≥ 2 prior lines of therapy, respectively.	1.2–4.2%
**Bi-specific T-cell engagers (BiTEs)**				
Blinatumomab [8]	Blincyto	BiTE → binds CD3 on T-cells and CD19 on B-cells	For children ≥1 year old with r/r Ph-negative CD19-positive BCP-ALL with MRD ≥0.1% (after >2 prior lines of therapy or r/r after allo-HSCT or r/r high-risk)	3.6–22%
**Tyrosine kinase inhibitors (TKIs)**				
Imatinib mesylate [9]	Gleevec, Glivec	Inhibits TK domain of ABL1, ABL2, KIT, KITLG, DDR1, DDR2, PDGFRA, PDGFRB AND CSF1R	For newly diagnosed children with Ph-positive ALL combined with chemotherapy and Ph-positive CML-CP or in blast crisis and accelerated phase in children not eligible for HSCT or after failure of IFN-α	0.4–9.7% as monotherapy for CML; 16.4–58.3% in combination with chemotherapy for Ph-positive ALL
Dasatinib [10]	Sprycel	Inhibits domains of ABL1, ABL2, Src family (SRC, LCK, YES1, FYN, HCK), KIT, EPHA2, and PDGFRB	For newly diagnosed children ≥1 year old with Ph-positive ALL combined with chemotherapy and Ph-positive CML-CP	0.9–1.1% as monotherapy for CML; 15.3–68.4% in combination with chemotherapy for Ph-positive ALL
Nilotinib [11]	Tasigna	Binds to and stabilizes the inactive conformation of the kinase domain of ABL proteins TK domain; Also inhibits PDGFRA, KIT, CSF1R, and DDR1	For children ≥1 year old with Ph-positive CML-CP at diagnosis or after other TKI failure	Up to 1.7%
Crizotinib [12]	Xalkori	Inhibits receptor TKs ALK (including fusion proteins EML4-ALK and NPM1-ALK), MET, ROS1, and MST1R	For children ≥1 year old with ALK-positive systemic ALCL	7.7–20.5%
Entrectinib [13]	Rozlytrek	Inhibits NTRK1, NTRK2, NTRK3, ROS1, ALK, JAK2, and TNK2 (including fusion proteins derived from *NTRK*, *ROS1*, and *ALK*)	For children ≥12 years old with r/r metastatic or unresectable solid tumors displaying *NTRK* fusion	2.3–8.3%
Larotrectinib [14]	Vitrakvi	Inhibits NTRK1, NTRK2, NTRK3, and TNK2		2–27.3% *
**Chimeric antigen receptor T-cells (CAR T-cells)**				
Tisagenlecleucel [15]	Kymriah	Reprogramming a patient’s own T-cells with a transgene encoding CAR → identification and elimination of CD19-positive cells	Children with r/r BCP-ALL	19–35.2% **

ABL = Abelson proto-oncogene non-receptor TK; ADC = antibody-drug conjugate; ALCL = anaplastic large cell lymphoma; ALK = anaplastic lymphoma receptor TK; ALL = acute lymphoblastic leukemia; allo-HSCT = allogeneic hematopoietic stem cell transplantation; AML = acute myeloid leukemia; B-AL = Burkitt leukemia (mature B-cell acute leukemia); BCP = B-cell precursor; BL = Burkitt lymphoma; BLL = Burkitt-like lymphoma; CAR = chimeric antigen receptor, comprised of a murine single-chain antibody fragment recognizing CD19 and fused to intracellular signaling domains from 4-1BB (CD137) and CD3 zeta; CD = cluster of differentiation; CML = chronic myelogenous leukemia; CP = chronic phase; DDR = discoidin domain receptor TK; DLBCL = diffuse large B-cell lymphoma; EML4 = echinoderm microtubule-associated protein-like 4; EPHA2 = ephrin type-A receptor 2; FYN = FYN proto-oncogene, Src Family TK; HCK = hemopoietic cell kinase proto-oncogene, Src Family TK; IFN = interferon; KIT = c-Kit proto-oncogene receptor TK or CD117; JAK2 = Janus kinase 2; KITLG = KIT ligand or stem cell factor (SCF) or mast cell growth factor; LCK = lymphocyte cell-specific proto-oncogene, Src family TK; MET = MET proto-oncogene, receptor TK or HGFR or C-Met; MRD = measurable residual disease; MST1R = macrophage stimulating 1 receptor or RON; NHL = non-Hodgkin lymphoma; NPM1 = nucleophosmin 1; NTRK = neurotrophic receptor TK or tropomyosin receptor kinase (Trk); PD-1 = programmed cell death 1 protein or CD279; PDGFR = platelet-derived growth factor receptor; Ph = Philadelphia chromosome or t(9;22)(q34;q11.2) translocation; PMBCL = primary mediastinal large B-cell lymphoma; ROS1 = ROS proto-oncogene 1, receptor TK; r/r = relapsed or refractory; SRC = SRC proto-oncogene non-receptor TK; TK = tyrosine kinase; TNK2 = TK non-receptor 2 or activated Cdc42-associated kinase 1 (ACK1); YES1 = YES proto-oncogene 1, Src Family TK; * = not possibly or probably treatment-related; ** = 21–48% according to SPC.

**Table 2:** Pooled odd ratios for grade ≥3 infections with targeted therapies in pediatric population treated for leukemias and lymphomas based on RCTs.

Targeted Therapy	Comparison	Odds Ratios for Grade ≥ 3 Infections
Rituximab (R)	Addition of R to revised NHL-BFM-95 protocol for BL [36]	No significant difference
	R-CHOP versus modified NHL-BFM-90 for DLBCL [25]	No significant difference
	Addition of R to standard chemotherapy for DLBCL [31]	No significant difference
	Addition of R to standard chemotherapy for CD20-positive ALL [43]	No significant difference
	Addition of R to modified FAB/LMB96 for stage III/IV B-NHL [29]	OR 2.73 (95% CI: 1.25 to 5.99; *p* = 0.006) during second induction for C1 group ^†^. No other significant differences during any other phase and across all groups.
	Addition of R to FAB/LMB96-based regimen for PMBCL [30]	No significant difference
	Addition of R to CCCG-B-NHL2015 protocol for B-NHL [37]	No significant difference
Gemtuzumab ozogamicin (GO)	Addition of GO to COG-AAML0531 regimen for de novo AML [48]	No significant difference
	Addition of chemotherapy to GO for r/r AML [49]	No significant difference
	Post-consolidation randomization to GO or no further therapy for AML (NOPHO-AML 2004) [50]	No significant difference
Pembrolizumab	N/A	
Blinatumomab (BLINA)	BLINA versus consolidation chemotherapy for Ph-negative, high-risk, first-relapse B-ALL [51]	No significant difference ^‡^
	Post-reinduction BLINA versus chemotherapy for high- and intermediate-risk first-relapse B-ALL [52]	OR 0.16 (95% CI: 0.08 to 0.35; *p* = 0.000002) after cycle 1, and OR 0.07 (95% CI: 0.07 to 0.17; *p* < 0.05) after cycle 2.
	BLINA versus InO for r/r BCP-ALL [53]	No significant difference
Imatinib	Addition of imatinib to chemotherapy for Ph-positive ALL [54]	No significant difference
	Imatinib plus chemotherapy for Ph-positive ALL compared with the same chemotherapy alone for Ph-negative ALL [55]	OR 10.24 (95% CI: 1.25 to 83.67; *p* = 0.015) during second reinduction. No other significant differences during any other phase.
	Addition of imatinib to chemotherapy for Ph-positive ALL [56]	No significant difference overall. As regards infection subcategories, significant difference for sepsis with OR 0.21 (95% CI: 0.04 to 1.07; *p* = 0.03) during induction only.
Imatinib versus dasatinib	For Ph-positive ALL [57]	No significant difference
Nilotinib	N/A	
Crizotinib	Crizotinib versus BV for de novo stage II-IV ALCL [58]	No significant difference
Entrectinib	N/A	
Larotrectinib	N/A	
Tisagenlecleucel	N/A	
Inotuzumab ozogamicin (InO)	InO versus BLINA for r/r BCP-ALL [53]	No significant difference
Brentuximab vedotin (BV)	Chemotherapy plus BV instead of vincristine for de novo high-risk classical HL [59]	No significant difference
	Addition of BV to chemotherapy for de novo ALCL [60,61]	No significant difference
	BV versus crizotinib for de novo stage II-IV ALCL [58]	No significant difference
Bortezomib	For de novo T-cell ALL and lymphoma [62]	OR 0.24 (95% CI: 0.16 to 0.38; *p* < 0.05) during induction; OR 0.26 (95% CI: 0.18 to 0.37; *p* < 0.05) during consolidation; OR 0.34 (95% CI: 0.24 to 0.47; *p* < 0.05) during delayed intensification.
Venetoclax	N/A	

BFM = Berlin-Frankfurt-Münster; CCCG = Chinese Children’s Cancer Group; COG = Children’s Oncology Group; CVAD = cyclophosphamide, vincristine, doxorubicin, and dexamethasone; FAB = French–American–British; LMB = lymphomes malins B; NOPHO = Nordic Society of Pediatric Hematology and Oncology; R-CHOP = cyclophosphamide, doxorubicin hydrochloride (hydroxy-daunorubicin), vincristine sulfate (Oncovin), and prednisone; R-ICE = rituximab, ifosfamide, carboplatin, and etoposide; ^†^ Group C1 patients = stage IV disease with marrow involvement >25% and CNS-negative; ^‡^ Significant higher rates of HGG in blinatumomab arm.

**Table 3:** Off-label targeted therapies in children with leukemia or lymphoma.

Targeted Therapy	Description	Off-Label Use	Grade ≥ 3 Infection Risk
**ALL**			
Inotuzumab ozogamicin (Besponsa) [176]	ADC recombinant humanized IgG4κ mAb linked to N-acetyl-gamma-calicheamicin → CD22	r/r Ph-positive BCP-ALL after failure of ≥1 TKI; r/r CD22-positive B-ALL	10.5% to 20%
Bortezomib (Velcade)	26S proteasome inhibitor	r/r ALL	16.2% to 45.5%
Also: bosutinib (Bosulif) and ponatinib (Iclusig)			
**AML**			
Venetoclax (Venclexta, Venclyxto) [177]	BCL2 inhibitor	r/r AML	15.3% to 50%
FLT3 inhibitors midostaurin (Rydapt) and gilteritinib (Xospata), and IDH inhibitor enasidenib mesylate (Idhifa)			
**HL**			
Brentuximab vedotin or SGN-35 (Adcetris) [178]	ADC chimeric IgG1 mAb linked to MMAE → CD30	r/r classical HL	3.4% to 5.3% for monotherapy; 3.3% to 15.6% when combined with chemotherapy
Also: nivolumab (Opdivo) and atezolizumab (Tecentriq)			
**NHL**			
Brentuximab vedotin or SGN-35 (Adcetris) [178]	As in HL above		
Also: ceritinib (Zykadia), atezolizumab (Tecentriq), nivolumab (Opdivo), ipilimumab (Yervoy), and ibritumomab (Zevalin)			

MMAE = monomethyl auristatin E.

**Table 4:** Risk of specific infectious complications and suggested prevention strategies.

Agent	Risk of HSV/VZV	Risk of PJP	Risk of HBV Reactivation	Risk of CMV Infection	Other Risk
Rituximab	Low	Low	Yes	Low	PML; Respiratory tract infections
GO	N/A (AML patients receive prophylaxis)				
Pembrolizumab	Only after irAEs	No	Yes	Only after irAEs	LTBI
Blinatumomab	Yes	Yes	Yes	Low	PML; HGG
Imatinib	Low	No	Yes	Low	
Dasatinib	Yes	No	Yes	Yes	
Nilotinib	Low	No	Yes	Low	
Tisagenlecleucel	Yes/Low	Yes	Yes	Yes	HGG; after CRS
InO	No	Low	Low	No	
BV	N/A (HSCT recipients receive prophylaxis)	N/A (HSCT recipients receive prophylaxis)	Yes	Yes	PML
Bortezomib	Low/Yes	Low	Low	Low	Respiratory tract infections
Venetoclax	No	No	No	No	

N/A = not available data.

## Supplementary Materials

The following supporting information can be downloaded at: https://www.mdpi.com/article/10.3390/cancers14205022/s1, Table S1: Comparison of reported infection rates with targeted therapies for leukemias and lymphomas between pediatric and adult population.
