# Supplementary material for: Infectious Complications of Targeted Therapies in Children with Leukemias and Lymphomas
Source: Cancers (Basel). 2022 Oct 14;14(20):5022. doi: 10.3390/cancers14205022 (PMC9599435; doi:10.3390/cancers14205022)
Supplement: Supplementary file 1 [file cancers-14-05022-s001.zip › cancers-1951786-supplementary.pdf]

Table S1. Comparison of reported infection rates with targeted therapies for leukemias and lymphomas between pediatric and adult population.

Agent	Pediatric patients (%)*	Adults (%)**
Rituximab (monotherapy)	2.9 – 4.2	5
Gemtuzumab ozogamicin	30.2 – 47 (monotherapy)<br>35.6 – 69.1 (combination)	32.8 (monotherapy)<br>41.2 – 77.9 (combination)
Pembrolizumab	1.2 – 4.2	7.3 – 27
Blinatumomab	3.6 – 22	9.7 – 22.6
Imatinib mesylate	0.4 – 9.7 (monotherapy)<br>16.4 – 58.3 (combination)	0.2 – 7 (monotherapy)<br>38 – 52 (combination)
Dasatinib	0.9 – 1.1	2 – 8
Nilotinib	≤1.7	≤1
Crizotinib	7.7 – 20.5	<1
Entrectinib	2.3 – 8.3	5.2 – 8.6
Larotrectinib	2 – 27.3	≤5
Tisagenlecleucel	19 – 35.2	16 – 48
Inotuzumab ozogamicin	10.5 – 20	5 – 28
Bortezomib	16.2 – 45.5	22 – 24
Venetoclax	15.3 – 50	19 – 64
Brentuximab vedotin	3.4 – 5.3	≤6

* Data from Tables 1 and 3 (original text)

* References: [1-15]

1. MabThera. Summary of product characteristics. European Medicines Agency (EMA). https://www.ema.europa.eu/en/documents/product-

information/mabthera-epar-product-information_en.pdf. Accessed 8 Oct 2022
2. Mylotarg. Summary of product characteristics. European Medicines Agency (EMA). https://www.ema.europa.eu/en/documents/product-information/mylotarg-epar-product-information_en.pdf. Accessed 8 Oct 2022
3. Keytruda. Highlights of prescribing information. Food and Drug Administration (FDA). www.fda.gov/medwatch. Accessed 8 Oct 2022
4. Blincyto. Summary of product characteristics. European Medicines Agency (EMA). https://www.ema.europa.eu/en/documents/product-information/blincyto-epar-product-information_en.pdf. Accessed 8 Oct 2022
5. Vitrakvi. Highlights of prescribing information. Food and Drug Administration (FDA). www.fda.gov/medwatch. Accessed 8 Oct 2022
6. Kymriah. Highlights of prescribing information. Food and Drug Administration (FDA). www.fda.gov/medwatch. Accessed 8 Oct 2022
7. Gleevec. Highlights of prescribing information. Food and Drug Administration (FDA). Accessed 8 Oct 2022
8. Sprycel. Highlights of prescribing information. Food and Drug Administration (FDA). Accessed 8 Oct 2022
9. Xalkori. Highlights of prescribing information. Food and Drug Administration (FDA). Accessed 8 Oct 2022 https://www.accessdata.fda.gov/drugsatfda_docs/label/2021/202570s030lbl.pdf. Accessed 8 Oct 2022
10. Besponsa. Highlights of prescribing information. Food and Drug Administration (FDA). www.fda.gov/medwatch. Accessed 8 OCT 2022
11. Tasigna. Highlight of prescribing information. Food and Drug Administration (FDA). Accessed 8 Oct 2022

12. Rozlytrek. Highlights of prescribing information. Food and Drug Administration (FDA). https://www.accessdata.fda.gov/drugsatfda_docs/label/2019/212725s000lbl.pdf Accessed 8 Oct 2022
13. Adcetris. Highlights of prescribing information. Food and Drug Administration (FDA). www.fda.gov/Safety/MedWatch. Accessed 8 Oct 2022
14. (2019) Venclexta. Highlights of prescribing information. Food and Drug Administration (FDA). www.fda.gov/medwatch. Accessed 8 Oct 2022
15. Velcade. Hoghlights of prescribing information. Food and Drug Administration (FDA). www.fda.gov/medwatch. Accessed 8 Oct 2022
